# Ability of Two Commercially Available Host-Targeted Technologies to Reduce Abundance of *Ixodes scapularis* (Acari: Ixodidae) in a Residential Landscape

**DOI:** 10.1093/jme/tjz046

**Published:** 2019-06-27

**Authors:** Robert A. Jordan, Terry L. Schulze

**Affiliations:** 1 Monmouth County Mosquito Control Division, 1901 Wayside Road, Tinton Falls, NJ 07724; 2 Terry L. Schulze, Ph.D., Inc., 9 Evergreen Court, Perrineville, NJ 08535

**Keywords:** Damminix Tick Tubes, SELECT TCS, *Ixodes scapularis*

## Abstract

Host-targeted technologies provide an alternative to the use of conventional pesticide applications to reduce the abundance of *Ixodes scapularis* Say, the vector for an array of tick-associated human diseases. We compared the ability of Damminx Tick Tubes (Damminix) and SELECT Tick Control System (Select TCS) bait boxes to control host-seeking *I. scapularis* nymphs in a wooded residential environment. Small mammals accepted and used Select TCS bait boxes with greater frequency compared to Damminix tubes over the course of the 2-yr trial. Nymphal tick infestation prevalence and intensity on captured mice and chipmunks provided no conclusive evidence of a treatment effect during May–June of both years. However, both treatments had a measurable effect on larval tick burdens in July–August and the magnitude of the effect was greater at the Select TCS-treated area and reflected the fact that Select TCS effectively treated chipmunks, while Damminix did not. Deployment of Damminix resulted in 27.6 and 20.3% control of questing nymphs in treated areas at 1 yr and 2 yr postintervention, while Select TCS bait boxes provided 84.0 and 79.1% control, respectively. The economics of residential tick control using these products in wooded residential landscapes is discussed.

Host-targeted technologies provide an alternative to wide-scale pesticide applications to control subadult *Ixodes scapularis* Say (Schulze et al. 2007). The first commercially available host-targeted product were Damminix Tick Tubes (hereafter Damminix), which consist of permethrin-treated cotton balls deployed in cardboard tubes. As designed, white-footed mice (*Peromyscus leucopus* Rafinesque) gather and use the cotton balls to use in nest construction, thereby treating themselves and any nest mates. Initial tests in Massachusetts reported dramatic decline in the number of ticks on mice and abundance of questing *I. scapularis* (Mather et al. 1987, 1988; Deblinger and Rimmer 1991). However, subsequent studies in residential areas of New York and Connecticut failed to replicate these results (Daniels et al. 1991; Stafford 1991, 1992). Despite showing significant reductions in immature *I. scapularis* infestation on white-footed mice due to Damminix treatment, these later studies also showed that infested mice continued to be recovered in treated areas and they were unable to demonstrate any effect on numbers of questing nymphs after treatment. Ginsberg (1992) also reported variable results from Damminix treatments, although numbers of ticks on white-footed mice were reduced, there was no uniform decrease in host-seeking *I. scapularis* nymphs following deployment.

Dolan et al. (2004) first demonstrated that deployment of fipronil using rodent bait boxes was able to substantially reduce larval and nymphal tick burdens on *P. leucopus* and numbers of host-seeking adult and nymphal *I. scapularis* on treated residential properties in Connecticut after 3 yr of treatment. This study led to the development of Maxforce Tick Management System (TMS) bait boxes, which were designed to treat small mammals as they contact a fipronil-treated wick while attempting to access baits. This concept has been shown effective in controlling subadult *I. scapularis* when used alone (Dolan et al. 2004, Schulze et al. 2017) or in concert with other tick control methods (Schulze et al. 2007, Williams et al. 2018). These bait boxes are currently marketed as SELECT Tick Control System (hereafter Select TCS).

We compared the relative ability of two commercially available host-targeted products, Damminix Tick Tubes and Select TCS bait boxes, to reduce the density of host-seeking nymphal *I. scapularis* within residential landscapes.

## Materials and Methods

### Study Areas

The 2-yr trial was conducted within a residential area located in Millstone Township, Monmouth County, New Jersey. All study sites were located within a mid-successional forest consisting of a 10- to 15-m canopy of oak (*Quercus prinus* L., *Q. rubra* L., and *Q. alba* L.) and American beech (*Fagus grandifolia* Ehrh.). The understory comprised oak, American holly (*Ilex opaca* Ait.) and sassafras (*Sassafras albidum* [Nutt.] Nees) above a pathy shrub layer of highbush blueberry (*Vaccinium corymbosum* L.), lowbush blueberry (*Vaccinium angustifolium* Ait.), and huckleberries (*Gaylussacia* spp.) (Schulze et al. 2007).

Recruitment of residential properties was accomplished through multiple mailings sent to selected property owners, based on size and configuration of suitable lots. Each of the 12 ± 1.0 ha properties consisted of lawn and landscaping immediately around the dwelling surrounded by woodland that covered from 25 to 75% of the property. None of the properties abutted another treated property and all properties were within 1,000 m of each other. Earlier surveys conducted in the vicinity of the study properties showed both *I. scapularis* and its small mammal hosts to be abundant (Schulze et al. 2005, 2007, 2017; Dolan et al. 2011). As in these previous studies, visually similar (species composition and stand age) forest in the nearby (within 1,000 m of treated residential properties) Assunpink Wildlife Management Area served as the untreated control site. Trapping at the untreated site was conducted in an area of ≈6 ha or equivalent to the area of the intervention sites.

### Damminix Tick Tubes

Damminix Tick Tubes (EcoHealth, Inc., Boston, MA) (hereafter Damminix) are 14.5 cm in length and 3.8 cm in diameter cardboard tubes that contain cotton balls treated with 7.4% permethrin. Prior to the study, the contents of 25 tubes from each carton were examined and shown to contain 15 cotton balls each. Twenty-four Damminix tubes were distributed on each of 6 study properties for each deployment. Host use of the product was determined at the end of each deployment period by counting the number of cotton balls within or nearby the tubes. A tick tube was deemed used when ≥ 1 cotton ball was unaccounted for. Irrespective of use, old tubes were retrieved and replaced with new tubes for each deployment.

### Select TCS Bait Boxes

Select TCS bait boxes (Tick Box Technology Corp., Norwalk, CT) are 19.05 × 13.97 × 6.35 cm child-resistant, injection-molded plastic boxes that house nontoxic bait attractants and a fipronil-treated felt wick assembly. Dolan et al. (2004) reported 42 d control of ticks on small mammals following a single treatment. Because of significant squirrel depredation observed in an earlier study (Schulze et al. 2007), we installed a two-piece, 0.032 gauge galvanized steel protective cover supplied by the manufacturer. The bait box and both sections of the protective cover were secured together at two opposite corners using 20.3 cm plastic cable ties (Thomas & Betts Corp., Memphis, TN). During each deployment, 24 bait boxes each were used on 4 properties, while 18 and 6 bait boxes were distributed on the remaining properties to reflect the amount of tick habitat. Use was defined as a ≥ 5.0 g reduction in overall weight of the bait box. At the end of each deployment, previously deployed bait boxes were replaced with new ones.

### Host-Targeted Interventions

Damminix tubes and Select TCS bait boxes were deployed in wooded areas of respective study properties for four separate 8-wk periods encompassing peak activity of *I. scapularis* nymphs (mid-May to mid-July) and larvae (mid-July to mid-September) in 2014 and 2015. Tubes or boxes were deployed along two concentric circles within the wooded portions of each property. The first circle of either tubes or bait boxes was deployed at ± 10 m from the lawn edge with a ± 20-m interval. We have previously found no difference in the efficacy of bait boxes when deployed at intervals of 10, 15, or 20 m (Schulze et al. 2007) and elected to use the more cost-effective 20 m interval for this trial, deploying Damminix tubes at the same interval for the sake of uniformity. When necessary, a second circle of tubes or boxes was placed ± 30 m from the lawn age with similar spacing. Wherever possible, tubes or boxes were deployed near areas where small mammals would likely forage or nest to maximize use, while few tubes or boxes were deployed in large expanses of open or sparse shrub layer.

### Small Mammal Trapping and Tick Burdens

To assess the cumulative effectiveness of Damminix and Select TCS treatments, we monitored burdens of subadult *I. scapularis* on live-captured small mammals from each of the treatment and control properties. We used Sherman (7.6- by 8.9- by 30.5-cm) nonfolding box traps (H.B. Sherman, Tallahassee, FL) baited with cotton balls and rolled oats to trap small mammals. We set 25 traps at each of the 12 intervention properties and 100 traps at the untreated area per day for a total of 800 trap nights per trapping event. Traps at treatment properties were deployed in 1–2 concentric rings around the house, targeting suitable small mammal habitat, but with a minimum of 15 m between traps. Traps at the untreated area were arrayed in 4 parallel transects, 20 m from roads, trails, or field edges, with a minimum of 15 m between traps. All traps were set by mid-afternoon on each trapping day and examined by 0900 h the follow day. Because eastern chipmunks, *Tamias striatus* L., were common in our study area, traps remained open during the day and checked periodically until late afternoon (Schulze et al. 2005). Small mammal captures were brought to our field station, anesthetized with isoflurane, examined for ticks, marked with individual metal ear tags (Monel Model 1005-1 or 1005-3, National Band and Tag Company, Newport, KY), and after recovering from the anesthesia, released at the capture site. Ticks obtained from small mammals were placed in individual vials containing 100% ethanol and marked with the corresponding ear tag number for subsequent species-level identification using published taxonomic keys (Keirans and Clifford 1978, Keirans and Litwak 1989, Keirans et al. 1996). Small mammals were not reprocessed if captured multiple times during a particular trapping event. Pre- and postdeployment nymphal tick burden data were obtained from two trapping events conducted during mid-May and mid-June, respectively, in 2014. Similarly, larval tick burdens were acquired from two additional trapping events in late July and late August 2014. Cumulative efficacy from subsequent deployments was determined from trapping performed in June and August 2015.

### Tick Collections

Efficacy was evaluated by monitoring density of host-seeking *I. scapularis* from 10 100-m transects on each group of treatment properties and the control site. Predeployment sampling was conducted in mid-May 2014 to coincide with the predeployment trapping event, while postdeployment assessments were performed in May–June 2015 and 2016. All collections were made using both dragging and walking survey techniques (Ginsberg and Ewing 1989, Schulze et al. 1997) conducted by the same field technicians between 0800 and 1200 h in an effort to minimize investigator or temporal bias (Schulze and Jordan 2003). Transects were sampled using 1-m^2^ light-colored corduroy cloth drags, worked alongside of each investigator using a 2-m rope handle attached to the ends of a wooden dowel at the leading edge of the drag. Any ticks found on drags or adhering to investigators clothing were removed at 20-m intervals identified, and returned to the transect (Schulze et al. 2007).

### Statistical Analyses

We used Mann–Whitney *U*-tests or Kruskal–Wallis multiple comparisons tests to compare tick burdens and numbers of questing ticks and contingency tables and χ^2^ or Fisher exact tests to compare infestation prevalence (percent of animals captured infested with at least 1 tick) before and after treatments (Sokal and Rohlf 1995). Percent control of questing ticks on treated properties was calculated using Henderson’s method: Percent control = 100 − (*T/U* × 100), where *T* and *U* are the mean after treatment/mean before treatment in treated and untreated properties, respectively (Henderson and Tilton 1955, Mount et al. 1976). All statistical tests were performed using Statistica analysis packages (StatSoft, Inc. 2005).

## Results

### Damminix Tick Tube Use

We recovered between 89.6 and 97.9% of the 144 Damminix tubes used at each of the four 8-wk deployments. Damminix use remained steady at between 20.3 and 25.6% over the first three deployments, but nearly doubled by the end of the fourth deployment (Table 1). Complete or partial damage to the Damminix label was observed on >75% of tubes for all four deployments.

### Select TCS Bait Box Use

Of the 120 Select TCS bait boxes used for each deployment, between 93.3 and 96.7% were recovered after 8 wk. However, all but one of the 25 missing boxes was eventually found during the winters of 2014 and 2015, with all demonstrating use. Bait box use increased incrementally after each deployment, reaching a maximum of 94.7% by the end of the study (Table 1).

During a total 26,880 deployment days, the protective metal cover was compromised only twice. In May 2014, one box had the protective cover removed and the box lid pried open. In May 2015, another box had the protective cover compromised, but the box remained intact. Damage was probably caused by raccoons (*Procyon lotor* L.).

### Small Mammal Trapping and Tick Burdens

White-footed mice and eastern chipmunks (*n* = 417), the primary *Borrelia burgdorferi* reservoir hosts in the study area (Schulze et al. 2005, 2017), comprised the vast majority of small mammal captures in both the treated and control site. The remaining captures (<2% of total) that were not processed included northern short-tailed shrew (*Blarina brevicauda* Say) and Virginia opossum (*Didelphis virginiana* Kerr). Mice comprised 70.0% of all captures during the 2-yr study. Overall, the ratio of mice to chipmunks was 120/47 at the Damminix sites, 71/63 at the Select TCS properties, and 101/15 at the control site.

During preintervention small mammals trapping in May 2014, infestation intensity (*I. scapularis* nymphs/capture) on mice and chipmunks did not differ significantly between treated and control areas (Kruskal–Wallis test, *H*_(2,17)_ = 2.21; *P* = 0.33) (Table 2) and the difference in infestation prevalence (proportion of captured small mammals with ticks) between the untreated area and either the Damminix (Fisher exact test [FET], *P* = 0.73) or Select TCS (*P* = 0.19) treatment areas was not statistically significant (Table 2). After the first 4 wk of interventions, trapping in June 2014 yielded nymphal tick burdens that were significantly lower in the untreated areas, but did not differ between the treatment areas (*H*_(2,41)_ = 1.00; *P* = 0.61) and infestation prevalence was similar in all areas.

Prior to the July 2014 deployments against larvae, larval burdens were higher, but not significantly different, in the untreated areas compared to either treatment area (*H*_(2,67)_ = 5.40; *P* = 0.07), however, infestation prevalence on mice and chipmunks was significantly reduced relative to the untreated area at both the Damminix (*P* = 0.02, FET) and Select TCS (*P* = 0.02, FET) properties (Table 2). This trend continued during trapping in mid-August at the Select TCS properties (*P* = 0.01, FET), but not at the Damminix properties (*P* = 0.17, FET). Infestation intensity did not differ between treatments (Mann–Whitney *U*_(23,21)_ =173.50, *P* = 0.09) and infestation prevalence was not significantly different between July and August at either the Damminix (*P* = 0.68, FET) or Select TCS (*P* = 0.23, FET) properties.

May 2015 trapping results showed that neither infestation prevalence (*P* = 0.58, FET) nor intensity of *I. scapularis* nymphs (*H*_(2,42)_ = 1.66; *P* = 0.44) on mice and chipmunks at the treated areas differed significantly from the control site (Table 3). After 4 wk of intervention (June 2015), infestation prevalence was significantly reduced at the Select TCS-treated properties, while tick burdens at the Damminix and untreated areas were equivalent (*H*_(2,66)_ = 8.74, *P* = 0.01; and Dunns test, *P* < 0.05). At the Select TCS site, infestation prevalence was reduced from that observed prior to deployment and relative to prevalence at the untreated site (*P* < 0.01 (FET) for both comparisons). Both prevalence (*P* < 0.01, FET) and intensity (*U*_(36,12)_ =106.50, *P* < 0.01) were lower at the Select TCS properties relative to Damminix properties.

During July 2015 trapping prior to deployments against larvae, infestation prevalence (*P* < 0.01, FET) and intensity (*H*_(2,62)_ = 25.39, *P* < 0.01) were both lower at the treated areas relative to the control site. Neither infestation prevalence (*P* = 0.14, FET) nor intensity (*U*_(25,16)_ =139.00, *P* = 0.06) differed between Damminix and Select TCS properties (Table 3). In August, following the final deployments against larval ticks, infestation prevalence (*P* < 0.01, FET) and intensity (*H*_(2,65)_ =26.176, *P* < 0.01) were both reduced at the treated areas compared to the untreated site. Neither infestation prevalence (*P* = 0.151, FET) nor infestation intensity (*U*_(26,15)_ =171.00, *P* = 0.516) differed between Damminix and Select TCS areas. Infestation prevalence at neither the Damminix (*P* = 0.49, FET) nor the Select TCS sites (*P* = 0.61, FET) differed from that observed in July and infestation intensity at neither treated area differed from that observed in July.

### Host-Seeking Ticks

Before the first deployments in May 2014, the density of host-seeking *I. scapularis* nymphs were statistically similar across treatment areas (Kruskal–Wallis test: (*H*_(2,26)_ = 0.56, *P* = 0.58) (Table 4). However, density of *I. scapularis* nymphs at the Select TCS treated properties decreased in both postdeployment years (*H*_(2,32)_ = 22.67; *P* < 0.01), representing 84.0% (2014) and 79.1% (2015) control of host-seeking ticks, while tick density on Damminix-treated properties remained similar to that observed from the untreated sites during the same period.

## Discussion

Based on the observed levels of reduction of questing *I. scapularis* nymphs, and to a lesser extent differences in tick burdens, Select TCS appears to provide much better control of questing ticks. This may be partially explained by the consistently lower use of Damminix. During 2014, 20.3 and 24.1% of Damminix tubes showed use by targeted small mammals during the first and second deployments, respectively. This rate of usage is considerably lower than previously reported (Daniels et al. 1991, Deblinger and Rimmer 1991; Stafford 1991, 1992). In contrast, 37.1 and 61.1% of Select TCS bait boxes were used during those respective deployments. Although also lower than previously described (Schulze et al. 2007, 2017), the rate of usage of bait boxes was more than double that of Damminix tubes. By the end of the fourth deployment (July–September 2015), use of Damminix (48.1%) was nearly double of that observed in earlier deployments, while use of Select TCS continued to increase, reaching a study high of 94.7%. The consistently lower use of Damminix compared to Select TCS may be attributable in part to the presence of other small mammals, most notably chipmunks, which do not harvest cotton and are often dominant in some residential landscapes (Schulze et al. 2005). However, mice composed the majority of small mammal captures at the treated properties during the 2 yr of this study so that host species composition was comparable between sites. The inability of Damminix to treat alternative hosts other than mice is a major drawback to its performance.

There was little evidence from 2014 deployments that either treatment had any substantial impact on nymphal tick burdens on small mammal captures. We observed a decline in infestation prevalence on captured mice and chipmunks after 4 wk at the Damminix sites in June 2014 and nymphal burdens did appear to be reduced at the Select TCS-treated area, relative to both the untreated and Damminix-treated areas, in spring 2015. However, the simultaneous decline in tick burdens at the untreated area from May to June suggested that some factor(s) unrelated to the treatment may have influenced the apparent decline in nymphal tick burdens at all areas. Results from trapping in July and August of both years suggested that continued deployments of both devices against both nymphs and larvae may have resulted in a cumulative effect on tick burdens. Summer tick burdens declined at both the Damminix and Select TCS properties over the 2 yr of the trial, relative to the untreated area, although low capture success sometimes prevented detection of a statistical difference. Consequently, evidence for an effect on small mammal tick burdens for either intervention is, at best, mixed.

Nevertheless, Select TCS bait boxes appeared to provide good control of questing nymphal ticks on treated residential properties. Schulze et al. (2017) also demonstrated significant reduction in the density of host-seeking *I. scapularis* nymphs, as well as significant declines in infestation intensity and prevalence, after Select TCS deployments in similar residential settings. In contrast, we showed no effect of Damminix treatment on questing ticks in the same areas, which is in concert with previous published trials of the product and appeared due, at least in part, to the presence of tick hosts other than mice using our treatment areas. These contrasting results, support the need for more research to elucidate the factors contributing to the efficacy of host-targeted tick control, including different habitat use by tick hosts in residential areas (Schulze et al. 2005), tick-host dynamics in multispecies host communities (Schmidt et al. 1999), potential for acaricide resistance as a result of repeated treatment by host-targeted devices (Ostfeld et al. 2006), and the possibility that host-targeted methods may actually result in the augmentation of host populations in treated areas, by providing either relief from tick burdens or an additional food resource (Ostfeld et al. 2006, White and Gaff 2018).

While Select TCS appeared to significantly reduce exposure to host-seeking *I. scapularis* nymphs in the treated areas, because this technology targets only immature ticks that have already acquired a host, any control of host-seeking ticks is not realized until many months or even years after initial deployment. As previously noted (Schulze et al. 2007, 2017), this delay in efficacy against host-seeking nymphs may result in substantial risk of exposure to *I. scapularis* nymphs well beyond the first deployment. Consequently, host-targeted methods such as Select TCS and Damminix may be most productive when used in an integrated tick management (ITM) approach in which targeted acaricide applications are made prior to deployment in order to provide rapid reduction of *I. scapularis* nymphs in high risk areas in an effort to minimize tick exposure until the host-targeted products become effective (Schulze et al. 2007, Williams et al. 2018).

Both Select TCS and Damminix were designed to control *I. scapularis* as the vector of *B. burgdorferi*. However, in a rapidly changing tick-borne disease landscape, with tick species other than *I. scapularis* playing an increasingly important role in the transmission of tick-borne pathogens (Stromdahl et al. 2012), we point out that neither of these devices is expected to have any effect on tick species which do not feed on the targeted small mammal hosts as immatures, such as the lone star tick (*Amblyomma americanum*) that is sympatric with *I. scapularis* in our study area (Schulze et al. 2005, 2006, 2011), typically occur at much higher densities than *I. scapularis* when sympatric (Schulze et al. 2006) and are suspected of playing a major role in reported tick-borne disease case rates (Egizi et al. 2017). Thus, to be effective in mitigating the risk of tick-borne disease in such areas, ITM programs must consider the possible presence of multiple tick vectors and include comprehensive preintervention surveys in prospective treatment areas in order to target all possible disease vectors (Schulze et al. 2009).

Apart from efficacy, our results illustrated some potential problems with both host-targeted methods. We observed that the Damminix regulatory label on over 76% of tubes was partially or totally destroyed in each of the 8-wk deployments, with ≈32% of tubes on two properties damaged after just 4 wk. Based on multiple observations, we attribute a significant portion of the damage to feeding by slugs (primarily *Limax maximus* L.). Since product labels rendered illegible by squirrel damage was a major contributing factor in the withdrawal of Maxforce TMS from the market, we suspect that this regulatory issue will need to be addressed in the future. Although the installation of the metal protective cover eliminated squirrel damage of Select TCS boxes, and appeared to reduce box loss over time (Schulze et al. 2017), a significant number of boxes (>50%) were disturbed or moved from their initial deployment location by nuisance wildlife over the course of the study. While this did not seem to affect use by target tick hosts, the potential loss of boxes missing at the end of deployment may be problematic. Most of the missing boxes at our study area were associated with two properties that adjoined a stream corridor that acted as a movement corridor for wildlife using the residential area and we believe that raccoons carried off the boxes. Where such interference by nontarget wildlife is a possibility, users should consider tethering boxes to metal stakes, as suggested by the manufacturer’s specifications, and using galvanized c-rings, rather than plastic cable ties to secure the protective covers.

Public acceptance and widespread use of any tick control method will also be based on factors such as product availability, cost, and safety. Our results suggest that Select TCS out-performed Damminix, with greater reductions in density of host-seeking nymphs. However, from the standpoint of the homeowner market, Damminix holds the clear advantage in both availability and cost. Select TCS bait boxes are only available through licensed pest management professionals (PMPs), whereas Damminix can be purchased and distributed by homeowners or PMPs. For each deployment, we used either 24 Damminix tubes or 24 Select TCS bait boxes to treat most of the properties. At the then current price of ± $75.00/case of 24 Damminix tubes, homeowners could personally treat their properties for about $150.00/year. In contrast, at a retail cost of ≈$45.00/unit, the cost for similar deployments of Select TCS bait boxes would exceed $2,100.00/year. Since surveys have shown that the majority of homeowners were not willing to spend > $100.00/year on tick control (Gould et al. 2008; R.A.J., unpublished data), the widespread acceptance and use of Select TCS may be cost-limited. From a safety perspective, the child-resistant design of Select TCS coupled with the protective metal cover made it the superior product. In most cases, the handling of Damminix tubes during deployment resulted in one or more cotton balls falling out of their tubes, requiring the user to manually replace them and, thus, repeatedly become exposed to permethrin. We also found cotton balls on the ground near the tubes at about one-third of the deployment sites. This resulted in further contact with permethin-treated cotton upon retrieval. Although the product labeling cautions against exposure to skin and eyes, contact with permethrin-treated cotton seems unavoidable.

Coupled with the inherent delays in efficacy that may result in significant exposure to ticks for extensive periods after initial deployment and relatively high cost, these potential drawbacks pose significant obstacles to the adoption of host-targeted technologies, used alone, as a tick control program that can achieve wide spread public acceptance. As part of an ITM program, it may be that efficacy of host-targeted tick control directed at rodent hosts must treat very large numbers (approaching 100%) of resident hosts over several consecutive years of treatment to achieve desirable levels of reduction in questing nymphal ticks (Mount et al. 1997, Eisen and Dolan 2016, Schulze et al. 2017). The necessary density of treatment devices is, in turn, dependent on any potential immigration of untreated hosts from adjacent untreated areas (Eisen and Dolan 2016). Without adequate knowledge of the composition of the host community in a treated area that accounts for seasonal and annual dynamics of host populations (including immigration and emigration rates into the treated area) (Dolan et al. 2004, Schulze et al. 2017, Williams et al. 2018), as well as better understanding of what proportion of various host populations are actually effectively treated by host-targeted interventions, we are unlikely to be able to conclusively assess the efficacy of these interventions. End users would benefit from additional research to determine a minimum Damminix tube or Select TCS box density that both treats sufficient small mammal hosts to be effective and makes the product more cost-effective for the homeowner (Schulze et al. 2017).

## Figures and Tables

**Table 1.** Summary of small mammal use of recovered Damminix Tick Tubes and Select TCS Bait Boxes deployed against subadult *I. scapularis* at Millstone Township, N.J. sites, May 2014–Sept. 2015

		Damminix			Select TCS		
Year	Deployment season	*n*	Tubes used (%)	Tubes empty (%)	*n*	Boxes used (%)	Boxes empty (%)
2014	May–June	138	22 (20.3)	19 (13.8)	116	43 (37.1)	13 (11.2)
	Aug.–Sep.	141	34 (24.1)	22 (15.6)	113	69 (61.1)	23 (20.4)
2015	May–June	129	33 (25.6)	8 (6.2)	112	98 (87.5)	52 (46.4)
	Aug.–Sep.	131	63 (48.1)	24 (18.3)	114	108 (94.7)	75 (65.8)

**Table 2.** Infestation prevalence (number infested and total number trapped) and intensity (mean number of ticks ± SE/captured animal) of *I. scapularis* subadults on live-trapped small mammals before and after intervention, May–August 2014

		Deployment vs nymphs						Deployment vs Larvae					
		May^1^			June			July^2^			August		
Treatment	Species	*n*	Prevalence (%)	Intensity	*n*	Prevalence (%)	Intensity	*n*	Prevalence (%)	Intensity	*n*	Prevalence (%)	Intensity
Untreated	*P. leucopus*	5	4 (80.0)	4.4 ± 2.5	6	3 (50.0)	1.5 ± 0.9	8	8 (100)	12.8 ± 5.8	9	7 (77.8)	4.8 ± 2.0
	*T. striatus*	0	-	-	2	1 (50.0)	6.8 ± 5.0	5	5 (100)	13.6 ± 10.7	3	3 (100)	3.3 ± 2.3
	All	5	4 (80.0)	4.4 ± 2.5	8	4 (50.0)	2.8 ± 1.5	13	13 (100)	13.2 ± 5.0	12	10 (83.3)	4.4 ± 1.6
Damminix	*P. leucopus*	5	4 (80.0)	5.0 ± 4.3	9	3 (33.3)	0.9 ± 0.8	18	9 (50.0)	6.1 ± 2.2	22	13 (59.1)	5.6 ± 2.3
	*T. striatus*	2	1 (50.0)	5.0 ± 5.0	4	4 (100)	3.0 ± 1.5	11	7 (63.6)	12.1 ± 6.8	1	1 (100)	1.0
	All	7	5 (71.4)	5.0 ± 3.1	13	7 (53.9)	1.4 ± 0.7	29	16 (55.2)	8.4 ± 2.9	23	14 (60.8)	5.2 ± 2.1
Select TCS	*P. leucopus*	1	0	0	6	3 (50.0)	2.0 ± 1.6	16	12 (75.0)	4.7 ± 1.3	18	7 (38.9)	2.5 ± 1.3
	*T. striatus*	4	2 (50.0)	1.0 ± 0.6	14	8 (57.1)	3.2 ± 1.5	9	2 (22.2)	0.2 ± 0.1	3	1 (33.3)	0.3 ± 0.3
	All	5	2 (40.0)	0.8 ± 0.5	20	11 (55.0)	2.9 ± 1.1	25	14 (56.0)	3.1 ± 0.9	21	8 (38.1)	2.2 ± 1.1

1 Results of small mammal trapping before initial deployment against nymphal ticks.

2 Results of small mammal trapping prior to second deployments against larval ticks.

**Table 3.** Infestation prevalence (number infested and total number trapped) and intensity (mean number of ticks ± SE/captured animal) of *I. scapularis* subadults on live-trapped small mammals before and after intervention, May–August 2015

		Deployment vs nymphs					Deployment vs Larvae					
		May^1^		June			July^2^			August		
Treatment	Species	*n*	Prevalence Intensity (%)	*n*	Prevalence (%)	Intensity	*n*	Prevalence (%)	Intensity	*n*	Prevalence (%)	Intensity
Untreated	*P. leucopus*	12	10 (83.3) 9.7 ± 2.7	18	15 (83.3)	2.3 ± 0.5	21	20 (95.2)	16.0 ± 3.1	23	23 (100)	15.0 ± 3.2
	*T. striatus*	4	1 (25.0) 1.8 ± 1.8	0	0	-	0	-	-	1	0	-
	All	16	11 (68.7) 7.7 ± 2.2	18	15 (83.3)	2.3 ± 0.5	21	20 (95.2)	16.0 ± 3.1	24	23 (95.8)	14.4 ± 3.1
Damminix	*P. leucopus*	8	5 (62.5) 4.8 ± 2.5	21	11 (52.4)	1.1 ± 0.3	12	3 (25.0)	4.0 ± 2.6	22	10 (45.5)	3.7 ± 1.9
	*T. striatus*	1	1 (100.0) 1.0	14	14 (100)	6.1 ± 1.5	10	8 (80.0)	6.4 ± 1.8	1	1 (100)	4.0
	All	9	6 (66.7) 4.4 ± 2.3	35	25 (71.4)	3.1 ± 0.7	22	11 (50.0)	5.3 ± 1.7	23	11 (47.8)	3.9 ± 1.9
Select TCS	*P. leucopus*	5	4 (80.0) 4.4 ± 2.7	4	1 (25.0)	1.0	9	2 (22.2)	1.4 ± 1.2	15	4 (26.7)	2.9 ± 2.0
	*T. striatus*	12	9 (75.0) 3.0 ± 1.3	9	3 (33.3)	0.8 ± 0.5	10	3 (30.0)	1.2 ± 0.9	3	0	1.0
	All	17	13 (76.5) 3.4 ± 1.2	13	4 (30.8)	0.9 ± 0.4	19	5 (26.3)	1.2 ± 0.9	18	4 (22.2)	2.3 ± 1.8

1 Results of small mammal trapping before second spring deployment against nymphal ticks.

2 Results of small mammal trapping prior to second summer deployment against larval ticks.

**Table 4.** Effects of host-targeted acaricide treatments against host-seeking nymphal *Ixodes scapularis* at Millstone Township, NJ, study areas

Treatment	2014	2015	2016	Kruskal–Wallis test
Untreated	9.9 ± 4.1*a*^1^	10.6 ± 4.1*a*	12.8 ± 5.2*a*	*H*_(2,32)_ = 2.11; *P* = 0.35
Select TCS	11.1 ± 5.9*a*	1.9 ± 1.1*b* (84.0%)^2^	3.0 ± 3.0*b* (79.1%)	*H*_(2,32)_ = 22.67; *P* < 0.01
Damminix	9.8 ± 4.1*a*	7.6 ± 4.1*a* (27.6%)	10.1 ± 3.9*a* (20.3%)	*H*_(2,32)_ = 3.18; *P* = 0.20

1 Values represent mean ticks ± SD/100-m^2^ (there were 10 randomly-assigned drags/treatment). There were no significant difference between treatments prior to the applications (Kruskal–Wallis test and Dunns post-hoc tests: *H*_(2,26)_ = 0.56, *P* = 0.58) prior to deployments. Means in the same row followed by the same letter are not significantly different.

2 Percent control, after Henderson’s equation: percent control = 100 −(*T/U* × 100), where *T* and *U* are the mean after treatment/mean before treatment in treated plots and untreated plots, respectively.
